# A Low-Cost and Environmentally Friendly Electrochemical Biosensor for the Determination of Estradiol

**DOI:** 10.3390/ma18132932

**Published:** 2025-06-20

**Authors:** Cecylia Wardak, Hubert Wólczyński, Szymon Malinowski, Beata Paczosa-Bator, Magdalena Wardak

**Affiliations:** 1 Department of Analytical Chemistry, Institute of Chemical Sciences, Faculty of Chemistry, Maria Curie-Sklodowska University in Lublin, Maria Curie-Sklodowska Sq. 3, 20-031 Lublin, Poland; wolczynski.hubert@gmail.com; 2 Department of Building Materials Engineering and Geoengineering, Faculty of Civil Engineering and Architecture, Lublin University of Technology, Nadbystrzycka St. 40, 20-618 Lublin, Poland; s.malinowski@pollub.pl; 3 Department of Analytical Chemistry and Biochemistry, Faculty of Materials Science and Ceramics, AGH University of Science and Technology, Mickiewicza St. 30, 30-059 Krakow, Poland; paczosa@agh.edu.pl; 4 Independent Public Health Care Center of the Ministry of Internal Affairs and Administration in Lublin, Grenadierów St. 3, 20-331 Lublin, Poland; magdawardak@gmail.com

**Keywords:** estradiol, enzyme biosensor, laccase, soft plasma polymerization, nanocomposite

## Abstract

Estradiol is a natural estrogen belonging to the group of natural steroid hormones. This paper presents new electrochemical biosensors—simple and low-cost tools for the determination of β-estradiol. The receptor layer of the sensor is the enzyme laccase, which was immobilized on the substrate surface using the soft plasma polymerization technique. This technique is innovative and environmentally friendly as it allows for the effective deposition of the enzyme onto unmodified and modified electrode substrates. Three types of substrates were used: an unmodified glassy carbon electrode and two electrodes modified with composite layers—multi-walled carbon nanotubes combined with CuO nanoparticles and multi-walled carbon nanotubes combined with carbon nanofibers, respectively. Biosensors modified with such materials have not been described previously. In the course of the study, electrochemical measurement conditions (composition, concentration and pH of the base electrolyte, sensor response time, and interference effects) were optimized, and sensor parameters were determined. It was found that the modification of the substrate electrode increased the sensitivity of the sensor by more than 25 times in both cases and led to a lower detection limit for the sensor modified with the carbon nanotubes/carbon nanofiber composite. The best performance was achieved with the sensor containing the carbon nanotube/carbon nanofiber composite layer, which showed a linearity range of 0.1–5 µM, a sensitivity of 7.32 ± 0.22 µA/µM, and a limit of quantification of 0.078 µM. The analytical utility of this biosensor was confirmed by its successful application in the determination of estradiol in pharmaceutical preparations and river water samples.

## 1. Introduction

Estradiol is a natural estrogen belonging to the group of natural steroid hormones, which, despite its natural origin, exhibits very potent biological effects, and even minimal concentrations of this hormone can induce serious changes in living organisms [1].

Estradiol is a component of many pharmaceutical preparations used in hormone replacement therapy. The presence of estradiol in the environment is a growing problem, as it enters from industrial and municipal wastewater and agricultural runoff. As a result, it is most often detected in surface waters such as rivers, lakes, and groundwater. These pollutants can lead to serious changes in aquatic ecosystems by upsetting their ecological balance and causing changes in the reproductive behaviour of aquatic organisms [2]. Estradiol-contaminated water also poses a risk to humans, especially when consumed. Such water can lead to endocrine disruption or an increased risk of certain cancers [3].

It is therefore necessary to develop effective methods for monitoring the estradiol content of both pharmaceutical preparations and natural waters. Traditional analytical techniques such as high-performance liquid chromatography (HPLC) and mass spectrometry (MS), despite their precision, are expensive and time-consuming. Electrochemical techniques are increasing in popularity due to their simplicity, sensitivity and low cost. They offer much faster and less expensive solutions that can be used in online conditions [4] or field analyses [5]. As an example, an electrochemical determination can be obtained in just a few minutes, and the equipment required is several times cheaper than that used in HPLC or MS techniques. The use of biosensors represents a further milestone in electrochemical detection methods [6]. Bioparticles such as enzymes can be used as a receptor layer, allowing for the selective determination of many analytes even at very low concentrations. With these developments, electrochemical sensors can be used for the real-time monitoring of water quality [7].

Laccases are among the oxidoreductases found in many living organisms, including higher plants, most fungi, and some bacteria [8]. Compared to other types of recognition bio-layers, the use of laccase enables electron transfer without additional cofactors in the quantification of many compounds as a result of their oxidation by molecular oxygen [9]. For this reason, biosensors based on the use of the biological enzyme precursor laccase are currently showing increasing popularity [10]. They are used both in environmental protection [11] and in many industries [12].

For biosensors, a key step in their preparation is the immobilization of the bioreceptor on the surface of the interface element. This operation must be carried out efficiently to maintain the biological activity of the receptor and ensure that the biosensor operates correctly, stably, and for as long as possible. Bio-functional coatings are currently deposited using wet chemical techniques and utilize multiple deposition steps. This involves the use of undesirable solvents, binders, couplers, and other chemicals that are expensive, hazardous, and unfriendly to produce [13,14]. In recent years, our research group has developed a new, innovative, cheap, one-step soft plasma polymerization (SPP) technique for applying receptor layers [6]. This method is based on corona discharge of cold atmospheric plasma close to room temperature. It is a simple and environmentally friendly method that allows for the efficient immobilization of receptor layers in a short one-step process, requiring no additional reagents [15,16].

Nanomaterials, especially carbon-based materials, are often used to modify electrodes for electrochemical sensors and biosensors [17]. These materials exhibit electrocatalytic properties and improve the charge exchange process at the electrode–sample interface, leading to an increased analytical signal and, consequently, better performance of the measurement procedure [18,19]. In recent years, composite and hybrid materials have become increasingly popular [20]. These materials are created from at least two different components in order to improve the performance of the individual components and/or to obtain new properties of the material being created. For sensing applications, the most common nanocomposites are obtained using carbon nanomaterials and other materials or nanomaterials, i.e., polymeric materials (especially conductive polymers) [21,22], ionic liquids [23,24,25], metal nanoparticles, and metal oxides [22,26,27]. Multi-component materials are also known [28,29]. In each case, the use of a nanocomposite brings tangible benefits.

This paper presents new biosensors for the determination of estradiol, which use the enzyme laccase as a receptor layer. The enzyme laccase was applied to the surface of a substrate electrode using the aforementioned soft plasma polymerization technology. A comparative study of sensors in which laccase was immobilized both on the surface of a bare glassy carbon electrode and on an electrode modified with composite materials, such as I-multiwalled carbon nanotubes and CuO nanoparticles, as well as II-multiwalled carbon nanotubes and carbon nanofibers, was carried out. Both composites exhibit desirable electrocatalytic properties, making them promising materials for the construction of electrochemical sensors and biosensors. CuONP/CNT nanocomposites, due to their higher charge-transfer efficiency [26] and greater hydrophobicity [27], proved to be a better material for glassy carbon electrode modification than CuONP and CNT separately in potentiometric sensors [26]. CNF/CNT composite is characterized by a unique structure, which results in a high surface-to-volume ratio, an increased number of active sites for adsorption and catalysis, and good conductivity of the material [30]. In addition, such composites show improved mechanical properties [31]. Due to these properties, carbon nanocomposites are promising for use as electrode modification materials in electrochemical sensors [32]. To the best of our knowledge, biosensors based on this type of composites have not yet been described in the literature.

## 2. Materials and Methods

### 2.1. Reagents

All chemicals used in experiments without further purification were of analytical grade. β-estradiol, sodium hydrogen phosphate (Na_2_HPO_4_), sodium dihydrogen phosphate (NaH_2_PO_4_), acetic acid (CH_3_COOH), sodium acetate (CH_3_COONa), nitric acid, glutaric acid (C_5_H_8_O_4_), lactose (C_12_H_22_O_11_), glucose (C_6_H_12_O_6_), glycine (C_2_H_5_NO_2_), mannitol (C_6_H_14_O_6_), saccharose (C_12_H_22_O_11_), starch, sodium chloride (NaCl), ascorbic acid (C_6_H_8_O_6_), multi-walled carbon nanotubes (CNT, diameter: 6–9 nm, length: 5 µm, purity ≥ 98%), carbon nanofibers (CNF 100 nm diameter, 20–200 µm length, purity > 99.9%), and copper oxide nanoparticles (CuONPs, particle size < 50 nm, purity > 99.5%) were purchased from Sigma Aldrich (St. Louis, MO, USA). Tetrahydrofuran (THF) and ethanol were purchased from Chempur (Piekary Śląskie, Poland).

The stock solution of estradiol at a concentration of 1 mM was prepared by the dissolution of an appropriate amount of this substance in a mixture of distilled water and ethanol (1:1). The working solutions of estradiol were prepared by diluting the stock solution as required.

The biological precursor in the form of the laccase enzyme was obtained from Cerrena unicolor C-139 and isolated according to the procedure described in reference [33]. The stock solution of laccase was obtained by the dissolution of liophylisate with a concentration of 178 mg/mL and activity of 186,000 nkat/L in 1 mL of freshly deionized water, and the working solution of laccase was prepared by diluting the stock solution (1:9). The concentration of laccase was measured spectrophotometrically using syringaldazine as a substrate.

**Figure 1:**
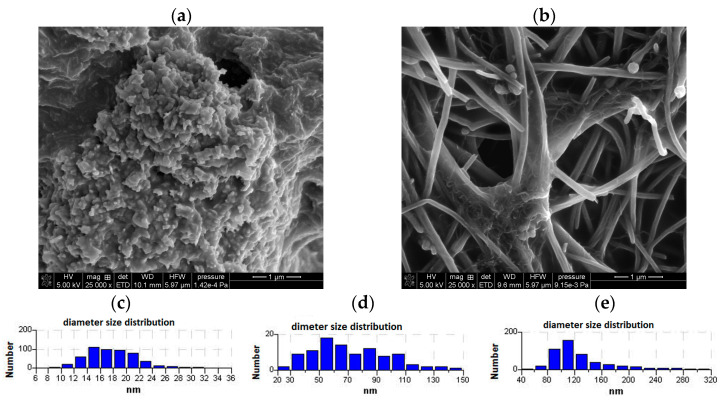
SEM images of CuONP/CNT nanocomposite (**a**) and CNF/CNT nanocomposite (**b**) and particle size distribution estimations of MWCNTs, (**c**) CuONPs (**d**) and CNFs (**e**).

The nanocomposites used to modify the glassy carbon electrode (GCE) were prepared by mixing 10 mg of MWCNTs with 10 mg of CuONPs (denoted as CuONP/CNT) or 10 mg of CNFs (denoted as CNF/CNT) and 10 mL of THF. The suspensions were then thoroughly homogenized in an ultrasonic bath for one hour and applied to the GCE surface by drop-casting. The nanocomposite formation was confirmed by scanning electron microscopy. SEM images of both composites are shown in Figure 1, where the homogeneous structure of both materials can be seen. The particle size distribution (Figure 1c–e) estimated from SEM measurements shows that in both cases, the particles do not aggregate and remain in the nano size after nanocomposite formation.

Samples of pharmaceutical preparations for estradiol determination were prepared in the following way: Ten tablets of each preparation were weighed and ground in a mortar, and then an amount equivalent to the weight of one tablet was taken. The contents were placed in a beaker, a distilled water and ethanol (1:1) mixture was added, and the beaker was placed in an ultrasonic bath for 15 min. Then, the solution was filtered and quantitatively transferred to a 25 mL flask and diluted with water.

River water samples were collected from the local river Czerniejówka into clean polypropylene vessels. They were analyzed after being filtered using a 0.45 μm Millipore membrane filter (Burlington, MA, USA). As no estradiol was found in the water analyzed, the samples were later spiked with 0.1 and 0.5 µM of estradiol. 

### 2.2. Biosensor Preparation

The biosensors were prepared by immobilizing the enzyme laccase onto an appropriately prepared substrate. An unmodified GCE and a GCE coated with a CuONP/CNT (50 µL suspension) or CNF/CNT (50 µL suspension) nanocomposite interlayer, respectively, were used as substrates. In each case, the GCE surface was carefully polished using fine-grained sandpaper and then with Al_2_O_3_ and wetted with distilled water.

**Figure 2:**
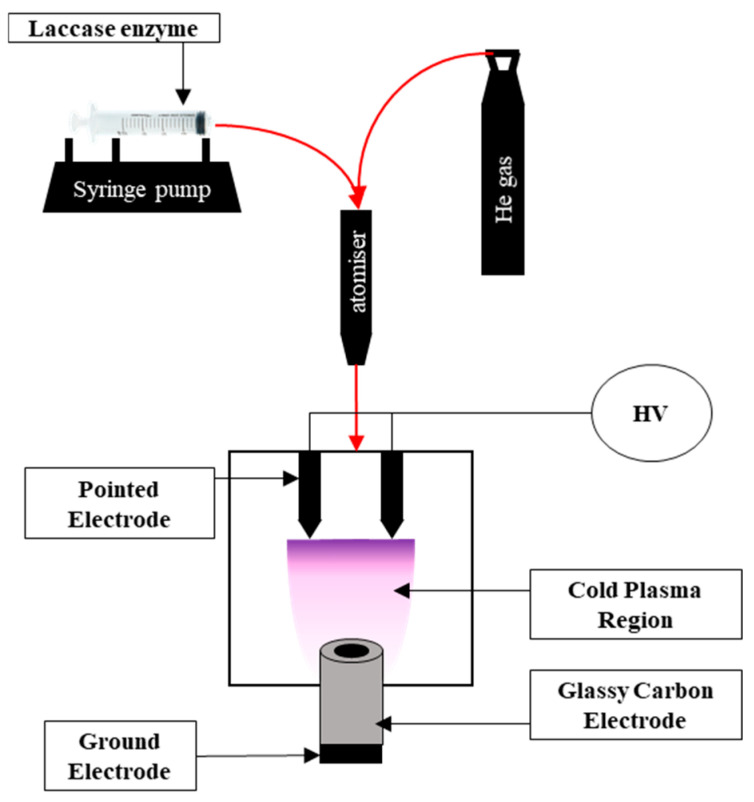
Scheme of set-up applied for laccase biorecognition layer deposition.

The biorecognition layer of laccase was applied using the SPP technique, which utilizes low-energy cold plasma corona discharges at temperatures not exceeding 40 °C under atmospheric pressure (Figure 2). The use of this type of discharge allows a combination of the presence of high-energy electrons, low temperature, and low energy density of the plasma jet. The enzyme solution is fed into the reaction zone in a stream of carrier gas (He in this case) in an atomized form, where it undergoes polymerization and crosslinking reactions (Figure 1). The SPP technique is an effective method for immobilizing enzymes, allowing for their efficient deposition on both the unmodified [6,16] GCE and the GCE modified with nanomaterials [15,34], while preserving the enzyme’s catalytic properties. The conditions of the laccase immobilization process affect the sensor parameters, as we have studied in detail in previous work. At an optimal deposition time of 30 s, the laccase receptor layer forms as large clusters are evenly distributed over the electrode surface, resulting in a sensor with the best performance compared to sensors obtained with shorter deposition times. Increasing the deposition time does not significantly affect the sensor’s performance [6]. The second parameter that significantly affects the properties of the sensor is the corona discharge potential. In this case, the best parameters were obtained for the sensor received at a potential of 3 kV [35]. It was also confirmed that the enzyme laccase remains active at approximately 60–80% after polymerization using the SPP technique [36]. The biosensors were prepared under optimal conditions (voltage, U = 3 kV; helium flow rate, V_He_ = 10 L/min; laccase solution flow rate, V_laccase_ = 200 µL/min; laccase deposition time, t = 30 s) obtained in previous studies [6,35]. In this way, three different biosensors were obtained, which will be referred to hereafter as GCE/Lac, GCE/(CuONP/CNT)/Lac, and GCE/(CNF/CNT)/Lac. Three sensors of each type were made and tested systematically over 3 weeks. Values were determined for linearity range, limit of detection (LOD), and sensitivity, and error bars on the calibration curves are given for the best sensor from four consecutive measurements. Data on the reproducibility of the sensor’s performance and its stability over time are given for three copies of a given sensor tested at the same time. For the unmodified GCE, the oxidation peak of estradiol was poorly formed, so this system was not included in the results.

### 2.3. Electrochemical Measurements

**Figure 3:**
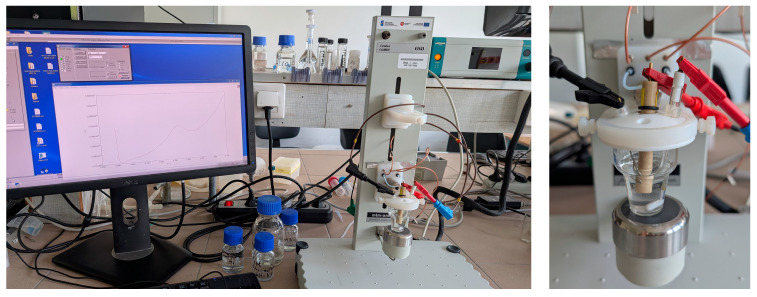
Photos of the measuring system and electrochemical cell with a set of three electrodes.

Electrochemical measurements were carried out using the differential pulse voltammetric (DPV) method using a µAutolab analyzer (Utrecht, The Netherlands). All measurements were carried out at room temperature (25.0–0.5 °C) in non-de-aerated, unstirred solution using an electrochemical cell volume of 10.00 mL and a conventional three-electrode system where the studied biosensor was applied as a working electrode, a Ag|AgCl (3 M KCl) electrode was used as the reference electrode, and a platinum electrode was used as an auxiliary electrode. First, the working electrode was polarized by applying a potential impulse at −1.4 V within 10 s. This pre-treatment step of the electrode is crucial for obtaining a stable signal as it allows for the removal of substances adsorbed during the previous measurement step from the surface of the biosensor. Next, after 5 s of solution equilibration, the potential was changed from 0.3 to 1.1 V, and DPV voltammograms were recorded. The measuring system and electrochemical cell with a set of three electrodes are presented in the photo (Figure 3).

## 3. Results and Discussion

### 3.1. Optimization of Supporting Electrolyte Solution

**Figure 4:**
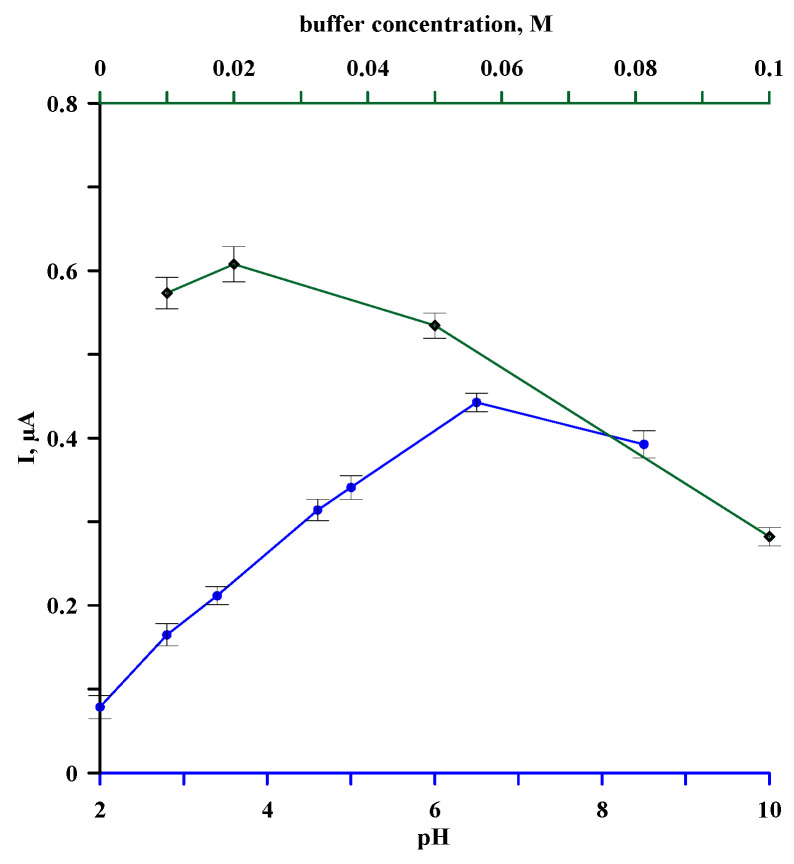
Effect of supporting electrolyte pH (blue curve) and phosphate buffer concentration (green curve) on electrochemical signal of estradiol oxidation recorded for GCE/Lac sensor.

The first stage of the study was to check the effect of the type and concentration of the base electrolyte solution on the recorded estradiol oxidation signal. For this purpose, DPV curves for GCE/Lac were recorded in 0.01 M solutions of the following electrolytes: nitric acid (pH = 2.0), acetate buffer (pH = 2.8; 3.4; 4.6; 5.0), phosphate buffer (pH = 6.5; 7.0), and ammonium buffer (pH = 8.5) containing 1.5 µM estradiol. The dependence of the peak current on the pH of the sample solution is shown in Figure 4 (blue curve). When analyzing this curve, it can be seen that the peak current for the oxidation of estradiol depends significantly on the pH and is the highest in the phosphate buffer at pH 6.5. The dependence of the GCE/Lac sensor signal on the concentration of this buffer in the concentration range of 0.1–0.01 M was then measured. The results of these measurements are shown in Figure 4 (green curve), from which it can be seen that the peak current increases as the buffer is diluted, reaching a maximum at a concentration of 0.02 M and then decreasing slightly. For further measurements, however, a buffer concentration of 0.05 M was chosen, for which the recorded signal is only slightly lower, and a higher buffer concentration will ensure that the buffer capacity of the electrolyte solution is sufficient to maintain the pH value during the analysis of real samples.

**Figure 5:**
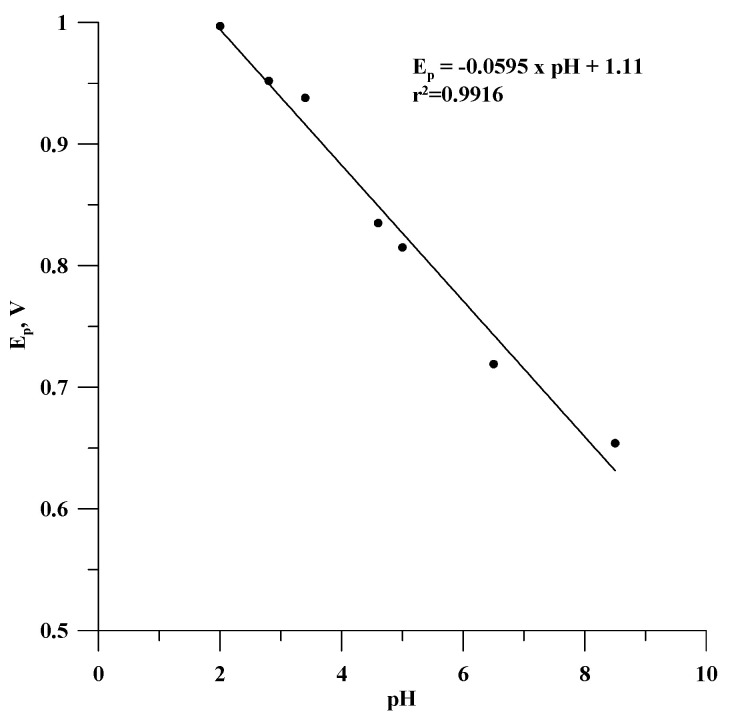
The dependence between the oxidation peak potential and determined pH values for the GCE/Lac sensor.

Based on the results of the measurements in solutions of different pH, the dependence of the oxidation peak potential of estradiol on the pH of the sample was determined (Figure 5). As can be seen, this relationship is linear and can be described by the equation Ep = −0.0595 pH + 1.11 with r^2^ = 0.9916. The slope (dEp/dpH) close to the Nernst value of −0.059 V/pH indicates that the oxidation reaction was proton-dependent, and the number of electrons exchanged was equal to the number of protons involved in the reaction. In this case, the oxidation of estradiol is associated with the transfer of one electron and one proton, which was also reported for the determination of this compound with another electrochemical analytical device [37,38]. This electrochemical behaviour of estradiol is due to its chemical nature. Estradiol is a very weak acid—its pKa is 10.71 [39]. In solutions with pH values lower than the pKa, the phenolic group is protonated, and the electrochemical oxidation of estradiol requires the donation of a proton.

### 3.2. Effect of Scan Rate and Potential Step

**Figure 6:**
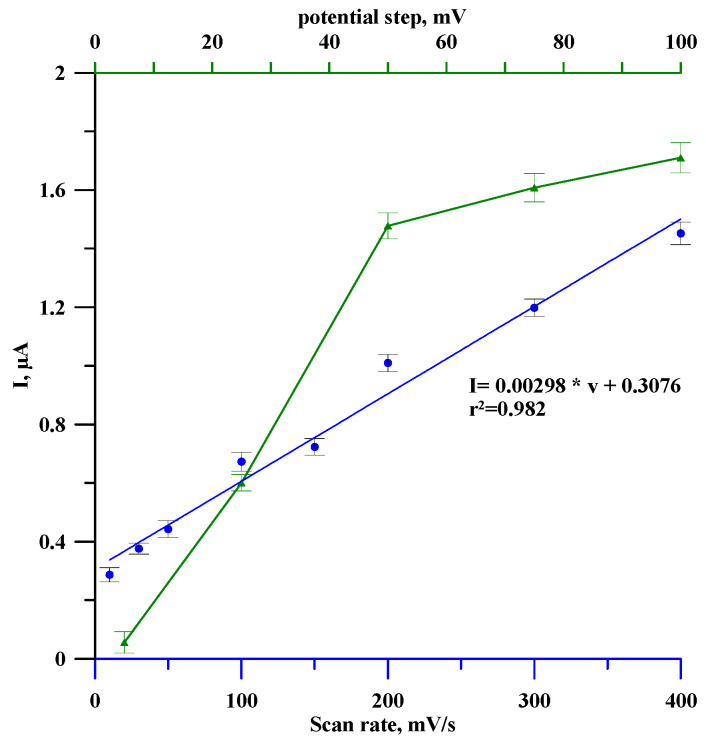
The dependence between the oxidation peak current potential and scan rate (blue line) and the determined potential step (green line) for the GCE/Lac sensor.

The next stage of the study was to examine the effect of electrochemical measurement parameters, such as the scan rate and potential step, on the peak current of estradiol oxidation. For this purpose, DPV curves were recorded for GCE/Lac in 0.05 M of phosphate buffer (pH = 6.5) containing 1.5 µM of estradiol under different conditions: a scan rate ranging from 10 to 400 mV/s with a constant and potential step of 50 mV and a potential step ranging from 5 to 100 mV with a scan rate of 100 mV/s. The obtained results are presented in Figure 6, where it can be seen that the recorded signal significantly depends on the measurement parameters. The oxidation peak current of estradiol oxidation increases dramatically with an increasing potential step up to a value of 50 mV, after which the increase is already small (green curve). A step value of 50 mV was selected as optimal.

Regarding the effect of the scan rate (blue curve), the recorded signal increased linearly with the increase in scan rate according to the equation I [µA] = 0.00298υ [mV/s] + 0.3076, with a correlation coefficient, r^2^, of 0.982. This linear dependency indicates that the electrode process of estradiol oxidation is surface-controlled [40].

**Figure 7:**
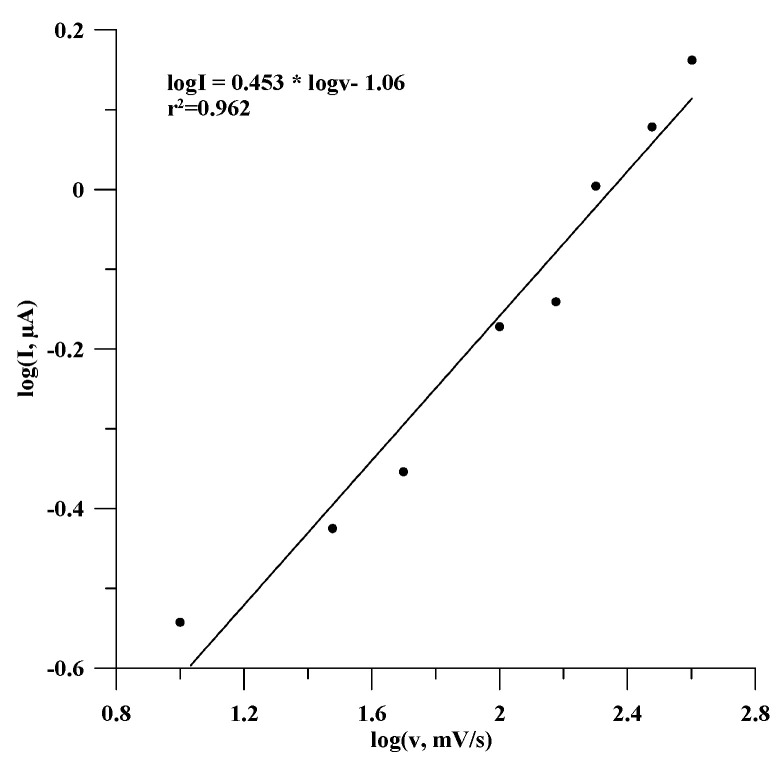
Dependence of decimal logarithm of peak current vs. decimal logarithm of scan rate.

The nature of the electrochemical process in estradiol oxidation can also be confirmed by the dependency of log(I) = f(logυ), presented in Figure 7. This dependency was linear and is described by the equation logI = 0.453 logυ − 1.06, with a correlation coefficient, r^2^, of 0.962. The slope of this linear relationship was 0.453, and it is close to the value of 0.5, so on this basis, it can be concluded that the process is a diffusion-controlled redox reaction [41].

### 3.3. Electrochemical Response of GCE/Lac, GCE/(CuONP/CNT)/Lac, and GCE/(CNF/CNT)/Lac

**Figure 8:**
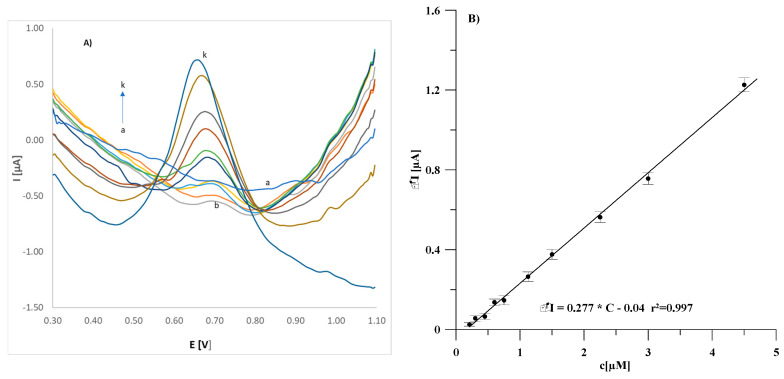
DPV voltammograms recorded for the GCE/lac sensor in an estradiol solution with concentrations ranging from 0 (a) to 5 µM (k) (**A**) and the corresponding calibration curve (**B**). The error bars represent the standard deviation from three measurements (*n* = 3).

Among the most important parameters of electrochemical sensors and biosensors that have a significant impact on their practical application are the linearity range of the calibration curve, the limit of quantification, and sensitivity. In the next stage of the study, the electrochemical behaviour of the studied sensors was determined in response to increasing concentrations of estradiol under the optimized conditions previously established. The influence of the estradiol concentration on the DPV curves recorded for GCE/Lac is presented in Figure 8A, and the corresponding calibration curve obtained from these results is shown in Figure 8B. As can be seen, the oxidation peak current of estradiol increased linearly in the range of concentrations from 0.2 to 4.5 µM according to the equation Δ I = 0.275 [estr] − 0.004 with a regression coefficient, r^2^, of 0.997. The limit of detection for this sensor was determined to be 0.061 µM, and the limit of quantification (LOQ), calculated as three times the LOD, was 0.18 µM.

**Figure 9:**
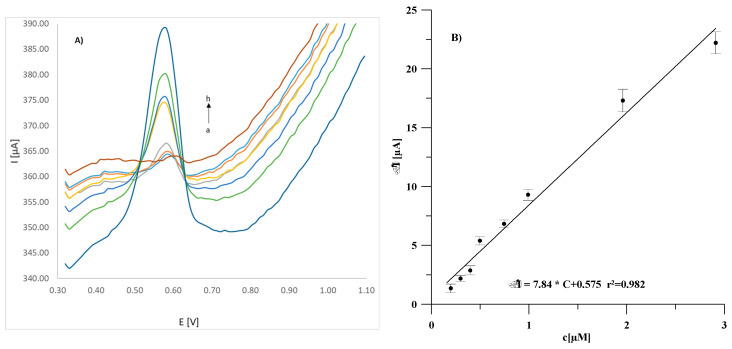
DPV voltammograms recorded for the GCE/(CuONP/CNT)/Lac sensor in an estradiol solution with concentrations ranging from 0 (a) to 3 µM (h) (**A**) and the corresponding calibration curve (**B**). The error bars represent the standard deviation from three measurements (*n* = 3).

**Figure 10:**
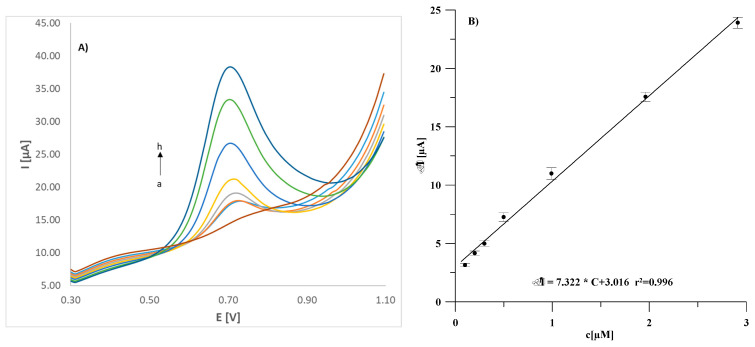
DPV voltammograms recorded for the GCE/(CNF/CNT)/Lac sensor in an estradiol solution with concentrations ranging from 0 (a) to 3 µM (h) (**A**) and the corresponding calibration curve (**B**). The error bars represent the standard deviation from three measurements (*n* = 3).

The same relationships determined for sensors obtained using modified substrates are shown in Figure 9 and Figure 10 for the GCE/(CuONP/CNT)/Lac and GCE/(CNF/CNT)/Lac biosensors, respectively. As can be seen, the modification of the electrode substrate with nanocomposites results in an increase in the peak current in both cases compared to the GCE/Lac biosensor. Both the GCE/(CuONP/CNT)/Lac and GCE/(CNF/CNT)/Lac biosensors showed more than 25 times higher sensitivity to estradiol, which was 7.84 ± 1.14 µA/µM for GCE/(CuONP/CNT)/Lac and 7.32 ± 0.22 µA/µM for GCE/(CNF/CNT)/Lac. The determined LOD values for these sensors were 0.058 µM and 0.026 µM for GCE/(CuONP/CNT)/Lac GCE/(CNF/CNT)/Lac, respectively. The LOQ values determined as three times the LOD value were 0.17 µM and 0.078 µM for GCE/(CuONP/CNT)/Lac and GCE/(CNF/CNT)/Lac, respectively. The increase in sensitivity of both sensors is certainly due to the increase in the active surface area of the electrode as a result of its modification with the nanocomposite. Unfortunately, in the case of the CNT/CuONPs composite-modified sensor, a significantly higher background current and poorer fit of the calibration curve points to the linear function (r^2^ = 0.982) were also observed. This was probably due to the structure of the CuONP/CNT nanocomposite and the non-specific adsorption of the analyte, which is often the reason for a high background current. For this reason, the GCE/(CNF/CNT)/Lac biosensor was selected for further testing.

### 3.4. Interference Studies for GCE/(CNF/CNT)/Lac Biosensor

To assess the selectivity of the tested biosensors toward estradiol in the presence of other chemical species, the influence of potentially interfering substances on the electrochemical signal of estradiol oxidation was investigated. To carry this out, the anodic peak currents in the estradiol solution at a concentration of 1.5 µM in 0.05 M of phosphate buffer at pH = 6.5 in the absence and presence of coexisting and potentially interfering substances at equal and 5-fold-higher concentrations were recorded. The potential interferents considered were glutamic acid, lactose, glucose, glycine, mannitol, saccharose, starch, ascorbic acid, and NaCl. Since one of the potential application areas of the proposed sensor is the analysis of real aqueous samples, we also decided to investigate how the organic matrix of such samples might influence the sensor’s signal. Humic and fulvic substances, which are the main constituents of organic matter in natural waters, were selected for the study. In this case, the oxidation signal of estradiol was recorded in the presence of 3 mg/L and 5 mg/L of the test substances, considering their average concentrations in river water, which range from 3.9 to 4.9 mg/L for humic acids and from 2.7 to 4.3 mg/L for fulvic acids. The sensitivity of the sensors tested to the presence of interfering substances was estimated based on the selectivity coefficient (SC), which was calculated using the equation SC = ((I_E+I_ − I_E_)/I_E_) × 100%, where I_E+I_ is the peak current generated for estradiol in the presence of coexisting substances, and I_E_ is the peak current generated for estradiol in the absence of coexisting substances. The obtained results are presented in Table 1, where it can be seen that in all cases, except for the humic and fulvic substances, the change in signal in the presence of the interferent did not exceed 5%. Therefore, the tested chemical species caused no significant interference in the electrode response current and will not affect the determination of estradiol using the GCE/(CNF/CNT)/Lac sensor. This is due to the selective catalysis of the oxidation reaction by the laccase molecules present in the receptor layer, resulting in an unclouded oestradiol signal even in the presence of substances potentially active in the potential range under investigation, e.g., ascorbic acid. Some problems may arise when analyzing water with a higher content of natural organic substances, which cause more than a 5% variation in the recorded signal. In such cases, it is necessary to remove these substances before electrochemical measurement, e.g., by using XAD-7 resin, as described in our previous study [42].

### 3.5. Reproducibility and Stability of GCE/(CNF/CNT)/Lac Biosensor

Key parameters of the sensor, especially for its practical, analytical application, particularly in series production, are its reproducibility and stability. The reproducibility and stability of the studied biosensor were evaluated by the determination of 1.5 µM of estradiol with four different freshly prepared GCE/(CNF/CNT)/Lac biosensors for 21 days. The relative standard deviation (RSD) obtained for the freshly prepared biosensors of 4.8% showed that the reproducibility of the fabrication of the laccase nanocomposite (CNF/CNT)-modified electrode is very good and only slightly deteriorates over time. The RSD value for the measured estradiol oxidation signal using the same sensors after 21 days was 7.73%. As for the stability of the sensor’s response, it was also quite satisfactory, showing close to 90% of the initial signal after three weeks of measurements.

### 3.6. Analytical Application of GCE/(CNF/CNT)/Lac Biosensor

To evaluate the analytical usefulness of the proposed bioelectrode, it was used for estradiol determination in pharmaceutical preparations named Estrofem 2 mg and Estrofem mite. They were purchased in the form of tablets at a local pharmacy and analyzed after preliminary preparation as described in the experimental section. The obtained results were compared to the declared content to calculate recovery values. Subsequently, the proposed biosensor was used for the analysis of natural water samples taken from the Czerniejówka river. The DPV curves recorded for the river water samples did not show any peaks of estradiol oxidation, which indicates that the concentration of this hormone was below the detection limit of the sensor. Therefore, the analyzed samples were spiked with estradiol at different concentration levels, and the content of this compound was determined using the standard addition method. The obtained results are shown in Table 2, where it can be seen that quantitative recovery was close to 100%, ranging between 95.2% and 99.0%. This means that the complex matrix of real pharmaceutical and environmental samples does not disturb the measurements and provides evidence that the proposed biosensor with a laccase biorecognition layer deposited using the soft plasma polymerization technique onto a substrate modified with CNF/CNT nanocomposite can be successfully used for estradiol determination in different real samples with satisfactory results.

## 4. Conclusions

This study demonstrates that the proposed biosensor developed by depositing a laccase biorecognition layer onto a CNF/CNT composite-modified substrate using the innovative soft plasma polymerization technique is an attractive tool for the electrochemical determination of estradiol. A comparison of the analytical performance of this sensor with that of other sensors previously described in prestigious journals is presented in Table 3. As shown, the proposed biosensor exhibits the highest sensitivity among the compared sensors. It also demonstrates lower detection limits and a relatively wide range of linearity of the calibration curve, making it comparable to other biosensors. Notably, the measurement procedure does not include an accumulation step, which allows for the detection limit to be lowered. An additional advantage of the proposed biosensor is its simple and quick preparation. Moreover, it is characterized by good stability and reproducibility. The measurement does not require the use of an agitator, and the time required for the measurement is short as it does not include additional steps such as accumulation, incubation in the sample solution, or the deoxygenation of the solution, making the sensor suitable for use under field conditions. Considering its good analytical performance and low manufacturing cost, the proposed biosensor has great application potential in multi-sensor systems, especially in the era of machine learning methods with a strong capability to process and classify large amounts of experimental data [43]. A certain limitation of the proposed sensor, typical of enzyme biosensors [44], is its relatively short lifespan due to enzyme degradation, necessitating the regeneration of the receptor layer. However, this process is neither lengthy nor complex.

## Figures and Tables

**Table 1:** Values of selectivity coefficient of proposed GCE/(CNF/CNT)/Lac biosensor.

Interferent	Selectivity Coefficient (SC), %	
	[E]:[I] = 1:1	[E]:[I] = 1:5
glutamic acid	2.81 ± 0.30	2.66 ± 0.41
lactose	3.11 ± 0.51	2.87 ± 0.55
glucose	2.58 ± 0.42	2.79 ± 0.48
glycine	4.53 ± 1.10	4.78 ± 0.90
mannitol	3.42 ± 0.41	3.21 ± 0.52
saccharose	1.11 ± 0.02	1.15 ± 0.03
starch	0.2 ± 0.11	0.40 ± 0.13
ascorbic acid	1.12 ± 0.21	1.10 ± 0.28
NaCl	0.10 ± 0.06	0.12 ± 0.04
humic substances	4.90 ± 1.11 ^a^	7.84 ± 1.90 ^b^
fulvic substances	3.71 ± 0.92 ^a^	5.14 ± 1.41 ^b^

[a] Data obtained in the presence of 3 mg/L of interfering substance. [b] Data obtained in the presence of 5 mg/L of interfering substance.

**Table 2:** Results of estradiol determination in real samples (n = 4).

Sample	Added, µM	Found, mg/Tablet; µM ^1^	Declared Content, mg/Tablet	Recovery%
Estrofem 2 mg	-	1.98 ± 0.021	2	99.0 ± 1.1
Estrofem mite	-	0.966 ± 0.018	1	96.6 ± 1.8
River water		-		-
River water + 0.1 µM	0.1 µM	0.0963 ± 0.0042		96.3 ± 4.2
River water + 0.5 µM	0.5 µM	0.476 ± 0.021		95.2 ± 4.2

^1^ mg/tablet for pharmaceutical preparation; µM for water samples.

**Table 3:** A comparison of the proposed biosensor with others previously reported in the literature.

	Linear Range, µM	Sensitivity µA/µM	LOD, µM	Accumulation Step	Additional Time (Accumulation/Incubation/Deaeration, min)	Ref.
MIPs/PtNPs/GCE	0.03–50	0.79	0.016	✓	14	[45]
BPIDS/GCE	0.1–10	0.19	0.050	✓	3	[46]
CuO/CPE	0.06–0.8	3.409	0.021	✗	nm	[47]
rGO-DHP/GCE	0.4–5.0	1.65	0.070	✓	2	[48]
CuThP/rGO/GCE	0.1–1	0.39	0.005	✗	nm	[49]
GCE/(CNF/CNT)/Lac	0.1–3	7.32	0.026	✗	**-**	This Work

✓-exist; ✗-not exists.

## Data Availability

The original contributions presented in this study are included in the article. Further inquiries can be directed to the corresponding author.

## References

[1] Baronti C., Curini R., D’Ascenzo G., Di Corcia A., Gentili A., Samperi R. (2000). Monitoring Natural and Synthetic Estrogens at Activated Sludge Sewage Treatment Plants and in a Receiving River Water. Environ. Sci. Technol..

[2] Huang B., Wang B., Ren D., Jin W., Liu J., Peng J., Pan X. (2013). Occurrence, Removal and Bioaccumulation of Steroid Estrogens in Dianchi Lake Catchment, China. Environ. Int..

[3] Combalbert S., Hernandez-Raquet G. (2010). Occurrence, Fate, and Biodegradation of Estrogens in Sewage and Manure. Appl. Microbiol. Biotechnol..

[4] Spychalska K., Zając D., Cabaj J. (2020). Electrochemical Biosensor for Detection of 17β-Estradiol Using Semi-Conducting Polymer and Horseradish Peroxidase. RSC Adv..

[5] Kidd K.A., Blanchfield P.J., Mills K.H., Palace V.P., Evans R.E., Lazorchak J.M., Flick R.W. (2007). Collapse of a Fish Population after Exposure to a Synthetic Estrogen. Proc. Natl. Acad. Sci. USA.

[6] Malinowski S., Wardak C., Jaroszyńska-Wolińska J., Herbert P.A.F., Panek R. (2018). Cold Plasma as an Innovative Construction Method of Voltammetric Biosensor Based on Laccase. Sensors.

[7] Baranwal J., Barse B., Gatto G., Broncova G., Kumar A. (2022). Electrochemical Sensors and Their Applications: A Review. Chemosensors.

[8] Dwivedi U.N., Singh P., Pandey V.P., Kumar A. (2011). Structure–Function Relationship among Bacterial, Fungal and Plant Laccases. J. Mol. Catal. B Enzym..

[9] Rodríguez-Delgado M.M., Alemán-Nava G.S., Rodríguez-Delgado J.M., Dieck-Assad G., Martínez-Chapa S.O., Barceló D., Parra R. (2015). Laccase-Based Biosensors for Detection of Phenolic Compounds. TrAC Trends Anal. Chem..

[10] Shahtouri M.G., Fooladi E., Marrazza G., Jahani M., Feizy J. (2025). Development of Laccase Electrochemical Biosensor for Detection of Dephostatin Using ZrO2/β-Cyclodextrin/Polyaniline Nanocomposite. Microchem. J..

[11] Chen H., Huang B., Han L. (2025). Enhanced Performance of Bacterial Laccase via Microbial Surface Display and Biomineralization for Portable Detection of Phenolic Pollutants. J. Hazard. Mater..

[12] Almeida L.C., Zeferino J.F., Branco C., Squillaci G., Morana A., Santos R., Ihalainen P., Sobhana L., Correia J.P., Viana A.S. (2025). Polynorepinephrine and Polydopamine-Bacterial Laccase Coatings for Phenolic Amperometric Biosensors. Bioelectrochemistry.

[13] Chen J., Leng J., Yang X., Liao L., Liu L., Xiao A. (2017). Enhanced Performance of Magnetic Graphene Oxide-Immobilized Laccase and Its Application for the Decolorization of Dyes. Molecules.

[14] Mei L.-P., Feng J.-J., Wu L., Zhou J.-Y., Chen J.-R., Wang A.-J. (2015). Novel Phenol Biosensor Based on Laccase Immobilized on Reduced Graphene Oxide Supported Palladium–Copper Alloyed Nanocages. Biosens. Bioelectron..

[15] Malinowski S., Wardak C., Pietrzak K. (2020). Effect of Multi-Walled Carbon Nanotubes on Analytical Parameters of Laccase-Based Biosensors Received by Soft Plasma Polymerization Technique. IEEE Sens. J..

[16] Malinowski S., Wardak C., Jaroszyńska-Wolińska J., Herbert P.A.F., Pietrzak K. (2020). New Electrochemical Laccase-Based Biosensor for Dihydroxybenzene Isomers Determination in Real Water Samples. J. Water Process Eng..

[17] Yohan R.K., Jagannathan M., Sivalingam G. (2025). The Role of Emerging Photoactive Nanostructures in Electrochemical Sensor Construction: Synthesis, Properties, Challenges, and Perspectives. J. Ind. Eng. Chem..

[18] Molaakbari E., Mostafavi A., Beitollahi H. (2015). Simultaneous Electrochemical Determination of Dopamine, Melatonin, Methionine and Caffeine. Sens. Actuators B Chem..

[19] Bounegru A.V., Dinu Iacob A., Iticescu C., Georgescu P.L. (2025). Electrochemical Sensors and Biosensors for the Detection of Pharmaceutical Contaminants in Natural Waters—A Comprehensive Review. Chemosensors.

[20] Wardak C., Pietrzak K., Morawska K., Grabarczyk M. (2023). Ion-Selective Electrodes with Solid Contact Based on Composite Materials: A Review. Sensors.

[21] Pietrzak K., Morawska K., Malinowski S., Wardak C. (2022). Chloride Ion-Selective Electrode with Solid-Contact Based on Polyaniline Nanofibers and Multiwalled Carbon Nanotubes Nanocomposite. Membranes.

[22] Lenar N., Piech R., Paczosa-Bator B. (2021). High Capacity Nanocomposite Layers Based on Nanoparticles of Carbon Materials and Ruthenium Dioxide for Potassium Sensitive Electrode. Materials.

[23] Wardak C., Morawska K., Paczosa-Bator B., Grabarczyk M. (2023). Improved Lead Sensing Using a Solid-Contact Ion-Selective Electrode with Polymeric Membrane Modified with Carbon Nanofibers and Ionic Liquid Nanocomposite. Materials.

[24] Wardak C., Pietrzak K., Grabarczyk M. (2021). Ionic Liquid-Multiwalled Carbon Nanotubes Nanocomposite Based All Solid State Ion-Selective Electrode for the Determination of Copper in Water Samples. Water.

[25] Morawska K., Wardak C. (2024). Application of Ionic Liquids in Ion-Selective Electrodes and Reference Electrodes: A Review. ChemPhysChem.

[26] Wardak C., Pietrzak K., Morawska K. (2023). Nanocomposite of Copper Oxide Nanoparticles and Multi-Walled Carbon Nanotubes as a Solid Contact of a Copper-Sensitive Ion-Selective Electrode: Intermediate Layer or Membrane Component–Comparative Studies. Appl. Nanosci..

[27] Morawska K., Malinowski S., Krawczyk J., Wardak C. (2024). Multi-Walled Carbon Nanotubes and Copper Oxide Nanoparticles Composite Used as Transducer Medias in Nitrate Ion-Selective Electrodes. J. Electrochem. Soc..

[28] Bagheri H., Hajian A., Rezaei M., Shirzadmehr A. (2017). Composite of Cu Metal Nanoparticles-Multiwall Carbon Nanotubes-Reduced Graphene Oxide as a Novel and High Performance Platform of the Electrochemical Sensor for Simultaneous Determination of Nitrite and Nitrate. J. Hazard. Mater..

[29] Liu B., Ouyang X., Ding Y., Luo L., Xu D., Ning Y. (2016). Electrochemical Preparation of Nickel and Copper Oxides-Decorated Graphene Composite for Simultaneous Determination of Dopamine, Acetaminophen and Tryptophan. Talanta.

[30] Niemiec B., Zambrzycki M., Piech R., Wardak C., Paczosa-Bator B. (2022). Hierarchical Nanocomposites Electrospun Carbon NanoFibers/Carbon Nanotubes as a Structural Element of Potentiometric Sensors. Materials.

[31] Navrotskaya A.G., Aleksandrova D.D., Krivoshapkina E.F., Sillanpää M., Krivoshapkin P.V. (2020). Hybrid Materials Based on Carbon Nanotubes and Nanofibers for Environmental Applications. Front. Chem..

[32] Smajdor J., Zambrzycki M., Paczosa-Bator B., Piech R. (2022). Use of Hierarchical Carbon Nanofibers Decorated with NiCo Nanoparticles for Highly Sensitive Vortioxetine Determination. Int. J. Mol. Sci..

[33] Mazur I., Rola B., Stolarczyk K., Nazaruk E., Bilewicz R., Rogalski J., Ohga S. (2015). The Large Scale Production of Cerrena Unicolor Laccase on Waste Agricultural Based Media. J. Fac. Agric. Kyushu Univ..

[34] Malinowski S., Wardak M., Wardak C. (2023). Effect of Modification of a Laccase-Based Electrochemical Biosensor with Carbon Nanotubes on Signal Separation of Dihydroxybenzene Isomers. Langmuir.

[35] Wardak C., Paczosa-Bator B., Malinowski S. (2020). Application of Cold Plasma Corona Discharge in Preparation of Laccase-Based Biosensors for Dopamine Determination. Mater. Sci. Eng. C.

[36] Malinowski S., Herbert P.A.F., Rogalski J., Jaroszyńska-Wolińska J. (2018). Laccase Enzyme Polymerization by Soft Plasma Jet for Durable Bioactive Coatings. Polymers.

[37] Zhan T., Tan Z., Tian X., Hou W. (2017). Ionic Liquid Functionalized Graphene Oxide-Au Nanoparticles Assembly for Fabrication of Electrochemical 2,4-Dichlorophenol Sensor. Sens. Actuators B Chem..

[38] Tao H., Wei W., Zeng X., Liu X., Zhang X., Zhang Y. (2009). Electrocatalytic Oxidation and Determination of Estradiol Using an Electrode Modified with Carbon Nanotubes and an Ionic Liquid. Microchim. Acta.

[39] Lewis K.M., Archer R.D. (1979). PKa Values of Estrone, 17β-Estradiol and 2-Methoxyestrone. Steroids.

[40] SALIMI A., MIRANZADEH L., HALLAJ R. (2007). Amperometric and Voltammetric Detection of Hydrazine Using Glassy Carbon Electrodes Modified with Carbon Nanotubes and Catechol Derivatives. Talanta.

[41] Bagheri H., Pajooheshpour N., Jamali B., Amidi S., Hajian A., Khoshsafar H. (2017). A Novel Electrochemical Platform for Sensitive and Simultaneous Determination of Dopamine, Uric Acid and Ascorbic Acid Based on Fe3O4SnO2Gr Ternary Nanocomposite. Microchem. J..

[42] Grabarczyk M., Fialek M., Wardak C. (2024). Use of Adsorption Properties of Resin for Water Sample Preparation in Voltammetric Determination of Se(IV) Using Bismuth Microelectrode. Molecules.

[43] Arano-Martinez J.A., Martínez-González C.L., Salazar M.I., Torres-Torres C. (2022). A Framework for Biosensors Assisted by Multiphoton Effects and Machine Learning. Biosensors.

[44] Wang X., Kong F., Liu Y., Lv S., Zhang K., Sun S., Liu J., Wang M., Cai X., Jin H. (2024). 17β-Estradiol Biosensors Based on Different Bioreceptors and Their Applications. Front. Bioeng. Biotechnol..

[45] Yuan L., Zhang J., Zhou P., Chen J., Wang R., Wen T., Li Y., Zhou X., Jiang H. (2011). Electrochemical Sensor Based on Molecularly Imprinted Membranes at Platinum Nanoparticles-Modified Electrode for Determination of 17β-Estradiol. Biosens. Bioelectron..

[46] Wang Z., Wang P., Tu X., Wu Y., Zhan G., Li C. (2014). A Novel Electrochemical Sensor for Estradiol Based on Nanoporous Polymeric Film Bearing Poly{1-Butyl-3-[3-(N-Pyrrole)Propyl]Imidazole Dodecyl Sulfonate} Moiety. Sens. Actuators B Chem..

[47] Antoniazzi C., de Lima C.A., Marangoni R., Spinelli A., de Castro E.G. (2018). Voltammetric Determination of 17β-Estradiol in Human Urine and Buttermilk Samples Using a Simple Copper(II) Oxide-Modified Carbon Paste Electrode. J. Solid. State Electrochem..

[48] Janegitz B.C., Dos Santos F.A., Faria R.C., Zucolotto V. (2014). Electrochemical Determination of Estradiol Using a Thin Film Containing Reduced Graphene Oxide and Dihexadecylphosphate. Mater. Sci. Eng. C.

[49] Moraes F.C., Rossi B., Donatoni M.C., de Oliveira K.T., Pereira E.C. (2015). Sensitive Determination of 17β-Estradiol in River Water Using a Graphene Based Electrochemical Sensor. Anal. Chim. Acta.

